# Supplementary material for: HIVprotI: an integrated web based platform for prediction and design of HIV proteins inhibitors
Source: J Cheminform. 2018 Mar 9;10:12. doi: 10.1186/s13321-018-0266-y (PMC5845081; doi:10.1186/s13321-018-0266-y)
Supplement: Supplementary file 2 — Additional file 2. Source code of HIVProtI web server. [file 13321_2018_266_MOESM2_ESM.zip › HIVprotI_Source-code/hivprotI/jsme/api_javadoc/export/client/JSME.html]

JSME


JavaScript is disabled on your browser.


Skip navigation links


- Package
- Class
- Tree
- Deprecated
- Index
- Help

- Prev Class
- Next Class

- Frames
- No Frames

- All Classes

- Summary:
- Nested |
- Field |
- Constr |
- Method

- Detail:
- Field |
- Constr |
- Method


export.client

## Class JSME

- java.lang.Object
- - export.client.JSME

- ---

    

  ```
  public class JSME
  extends java.lang.Object
  ```

- - ### Constructor Summary

    Constructors

    Constructor and Description

    `JSME(java.lang.String htmlId)`

    `JSME(java.lang.String htmlId,
    JavaScriptObject parameterJso)`

    `JSME(java.lang.String htmlId,
    java.lang.String width,
    java.lang.String height)`

    `JSME(java.lang.String htmlId,
    java.lang.String width,
    java.lang.String height,
    JavaScriptObject parameterJso)`
  - ### Method Summary

    All Methods Static Methods Instance Methods Concrete Methods Deprecated Methods

    | Modifier and Type | Method and Description |
    | --- | --- |
    | `void` | `addClickHandler(JsClosureJsme jsEventHandler)` Add a click handler to a JSME instance. |
    | `void` | `addMouseDownHandler(JsClosureJsme jsEventHandler)` Add a mouse down handler to a JSME instance. |
    | `void` | `addMouseOutHandler(JsClosureJsme jsEventHandler)` Add a mouse out handler to a JSME instance. |
    | `void` | `addMouseOverHandler(JsClosureJsme jsEventHandler)` Add a mouse over handler to a JSME instance. |
    | `void` | `alert(java.lang.String message)` Display an alert box with the look and feel of JSME |
    | `void` | `clear()` Same effect as clicking on the clear icon: clear the molecular drawing area |
    | `void` | `deferredRepaint()` Repaint the applet after the browser event loop returns. |
    | `java.lang.String[]` | `getAlldefinedCallBackNames()` Return an array containing all possible callback names. |
    | `JsArrayString` | `getAllGraphicsString()` Returns all graphics representations used in the applet, either a SVG or a VML string This is an advanced feature. |
    | `JavaScriptObject` | `getCallBack(java.lang.String callbackName)` Generic callback |
    | `int` | `getCreationIndex()` The JSME class keeps tract of the number of instance that have been created. |
    | `java.lang.String` | `getHelpURL()` |
    | `double` | `getMenuScale()` |
    | `boolean` | `getMolecularAreaAntiAlias()` Return the antialias setting for the molecular drawing area |
    | `java.lang.String` | `getMolecularAreaGraphicsString()` Returns the graphics representation used in the molecular area, either a SVG or a VML string This is an advanced feature. |
    | `float` | `getMolecularAreaLineWidth()` |
    | `double` | `getMolecularAreaScale()` |
    | `java.lang.String` | `getNotifyStructuralChangeJSfunction()` Deprecated. |
    | `int` | `getOffsetHeight()` Gets the object's offset height in pixels. |
    | `JsArrayInteger` | `getOffsetSize()` Gets the object's offset width and height in pixels. |
    | `int` | `getOffsetWidth()` Gets the object's offset width in pixels. |
    | `com.google.gwt.user.client.Element` | `getParentContainer()` Return the parent DOM element that contains myself. |
    | `java.lang.String` | `getPasteLabel()` See `setPasteLabel(String)` |
    | `java.lang.String` | `getPrePasteJSfunction()` |
    | `java.lang.String` | `getWebSearchInchiKeyBaseUrl()` |
    | `void` | `initializationError(java.lang.String message)` |
    | `boolean` | `isDepictMode()` |
    | `boolean` | `isVisible()` Returns whether the given element is visible in a way consistent with `setVisible(boolean)`. |
    | `java.lang.String` | `jmeFile()` |
    | `java.lang.String` | `molFile(boolean isV3000)` |
    | `java.lang.String` | `nonisomericSmiles()` |
    | `void` | `options(java.lang.String options)` options is a comma separated value list - same as original JME |
    | `boolean` | `read_MOL_SDF_RXN(java.lang.String s)` Read a molecular structure encoded in MOL or SDF format, or a reaction encoded in RXN format. |
    | `void` | `readGenericMolecularInput(java.lang.String s)` |
    | `void` | `readMolecule(java.lang.String s)` Read a molecular structure encoded in JME format |
    | `void` | `readMolFile(java.lang.String s)` Read a molecular structure encoded in MOL format, or a reaction encoded in RXN format |
    | `void` | `repaint()` Redraw the whole applet if it is visible |
    | `static int` | `replaceAllAppletsByJSME()` Scan the DOM for DIV's containing an attribute code="JME.class" and install a running JSME. |
    | `static int` | `replaceAllAppletsByJSME(JsClosure jsFunctionWithJsmeInstanceArg)` Scan the DOM for DIV's containing an attribute code="JME.class" and install a running JSME. |
    | `void` | `reset()` |
    | `void` | `resetAtomColors(int molIndex)` Reset background colors |
    | `void` | `setAction(int action)` select one of the menu action without having to click on the menu. |
    | `void` | `setAfterDepictEditToggleEventHandler(JsClosureJsme jsEventHandler)` |
    | `void` | `setAfterPasteCallback(JavaScriptObject callbackFunction)` Deprecated. |
    | `void` | `setAfterStructureModifiedCallback(JavaScriptObject callbackFunction)` Deprecated. |
    | `void` | `setAntialias(boolean onOff)` Set globally antialias |
    | `void` | `setAtomBackgroundColors(int molIndex, java.lang.String atomAndColorCSV)` |
    | `void` | `setAtomMolecularAreaFontSize(float fs)` |
    | `void` | `setAtomToHighLight(int molIndex, int atomIndex)` |
    | `void` | `setBeforePasteCallback(JavaScriptObject callbackFunction)` Deprecated. |
    | `void` | `setCallBack(java.lang.String callbackName, JavaScriptObject callbackFunction)` Generic call back. |
    | `void` | `setHeight(java.lang.String width)` Sets the object's height. |
    | `void` | `setHelpURL(java.lang.String url)` set the url to display the JSME help page. |
    | `void` | `setMenuScale(double scale)` |
    | `void` | `setMolecularAreaAntiAlias(boolean molecularAreaAntiAlias)` Set antialias for the molecular drawing area |
    | `void` | `setMolecularAreaLineWidth(float molecularAreaLineWidth)` Line width in pixel |
    | `void` | `setMolecularAreaScale(double scale)` |
    | `void` | `setNotifyAtomHighLightChangeJSfunction(java.lang.String notifyAtomHighLightJSfunction)` Deprecated. |
    | `void` | `setNotifyStructuralChangeJSfunction(java.lang.String notifyStructuralChangeJSfunction)` Deprecated. |
    | `void` | `setPasteLabel(java.lang.String pasteLabel)` Set the label of the context popup menu for pasting string, instead of using the default label. |
    | `void` | `setPrePasteJSfunction(java.lang.String prePasteJSfunction)` Deprecated. |
    | `void` | `setSize(int width, int height)` Sets the object's size. |
    | `void` | `setSize(int width, int height, boolean resizeParent)` Sets the object's size. |
    | `void` | `setSize(java.lang.String width, java.lang.String height)` Sets the object's size. |
    | `void` | `setSize(java.lang.String width, java.lang.String height, boolean resizeParent)` Sets the object's size. |
    | `void` | `setSubstituent(java.lang.String s)` Select the next FG to be used. |
    | `void` | `setTemplate(java.lang.String templateAsJmeMol, java.lang.String templateName)` Provide a template, a molecular fragment with one marked atom, in JME format. |
    | `void` | `setVisible(boolean visible)` Shows or hides the given element. |
    | `void` | `setWebSearchInchiKeyBaseUrl(java.lang.String webSearchInchiKeyBaseUrl)` Set the base url for inchi key search. |
    | `void` | `setWidth(java.lang.String width)` Sets the object's width. |
    | `void` | `showInfo(java.lang.String message)` Display a message in the bottom bar. |
    | `java.lang.String` | `smiles()` |

    - ### Methods inherited from class java.lang.Object

      `equals, getClass, hashCode, notify, notifyAll, toString, wait, wait, wait`

- - ### Constructor Detail


    - #### JSME

      ```
      public JSME(java.lang.String htmlId,
                  java.lang.String width,
                  java.lang.String height)
      ```


    - #### JSME

      ```
      public JSME(java.lang.String htmlId)
      ```


    - #### JSME

      ```
      public JSME(java.lang.String htmlId,
                  java.lang.String width,
                  java.lang.String height,
                  JavaScriptObject parameterJso)
      ```


    - #### JSME

      ```
      public JSME(java.lang.String htmlId,
                  JavaScriptObject parameterJso)
      ```

      Parameters:
      :   `htmlId` -
      :   `parameterJso` -
  - ### Method Detail


    - #### getAllGraphicsString

      ```
      public JsArrayString getAllGraphicsString()
      ```

      Returns all graphics representations used in the applet, either a SVG or a VML string
      This is an advanced feature.

      Returns:
      :   an array of strings that can be used in a DOM element innerHTML


    - #### getMolecularAreaGraphicsString

      ```
      public java.lang.String getMolecularAreaGraphicsString()
      ```

      Returns the graphics representation used in the molecular area, either a SVG or a VML string
      This is an advanced feature.

      Returns:
      :   a string that can be used in a DOM element innerHTML


    - #### getWebSearchInchiKeyBaseUrl

      ```
      public java.lang.String getWebSearchInchiKeyBaseUrl()
      ```

      Returns:
      :   the web search InchiKey base Url
          See also see `setWebSearchInchiKeyBaseUrl(String webSearchInchiKeyBaseUrl)`


    - #### setWebSearchInchiKeyBaseUrl

      ```
      public void setWebSearchInchiKeyBaseUrl(java.lang.String webSearchInchiKeyBaseUrl)
      ```

      Set the base url for inchi key search. For instance: "http://www.google.com/search?q="

      Parameters:
      :   `webSearchInchiKeyBaseUrl:` - the web search InchiKey base Url to set
          Note: alternatively, one can replace the web search by a custom function call.
          See `setCallBack(String,JavaScriptObject)`
          To find the inchi key search callback name, see `getAlldefinedCallBackNames()`
          See also see `getWebSearchInchiKeyBaseUrl()`


    - #### getMolecularAreaScale

      ```
      public double getMolecularAreaScale()
      ```

      Returns:
      :   the scale of the molecular area


    - #### setMolecularAreaScale

      ```
      public void setMolecularAreaScale(double scale)
      ```


    - #### getMenuScale

      ```
      public double getMenuScale()
      ```


    - #### setMenuScale

      ```
      public void setMenuScale(double scale)
      ```


    - #### getCreationIndex

      ```
      public int getCreationIndex()
      ```

      The JSME class keeps tract of the number of instance that have been created. Each instance has a copy of its creation count, which can be used as a index.
      The first instance has an index equal to 0.

      Returns:
      :   the creation index.


    - #### getParentContainer

      ```
      public com.google.gwt.user.client.Element getParentContainer()
      ```

      Return the parent DOM element that contains myself. This is equivalent to using document.getElementById("id") where "id" is the id of the element that was used to create the JSME instance.


    - #### initializationError

      ```
      public void initializationError(java.lang.String message)
      ```


    - #### setAntialias

      ```
      public void setAntialias(boolean onOff)
      ```

      Set globally antialias

      Parameters:
      :   `onOff` -


    - #### options

      ```
      public void options(java.lang.String options)
      ```

      options is a comma separated value list - same as original JME

      Parameters:
      :   `options` -


    - #### readMolecule

      ```
      public void readMolecule(java.lang.String s)
      ```

      Read a molecular structure encoded in JME format

      Parameters:
      :   `s` -


    - #### readMolFile

      ```
      public void readMolFile(java.lang.String s)
      ```

      Read a molecular structure encoded in MOL format, or a reaction encoded in RXN format

      Parameters:
      :   `molecule` -


    - #### read\_MOL\_SDF\_RXN

      ```
      public boolean read_MOL_SDF_RXN(java.lang.String s)
      ```

      Read a molecular structure encoded in MOL or SDF format, or a reaction encoded in RXN format.
      If the input is a SDF with more than one structure, then all SDF entries will be stored in the internal SDF buffer.
      Return true if successful or false otherwise.
      TODO: fix bug with paste event - infinite recursion

      Parameters:
      :   `s` -

      Returns:
      :   success


    - #### readGenericMolecularInput

      ```
      public void readGenericMolecularInput(java.lang.String s)
      ```


    - #### molFile

      ```
      public java.lang.String molFile(boolean isV3000)
      ```

      Parameters:
      :   `isV3000` -

      Returns:
      :   the MOL or the RXN


    - #### smiles

      ```
      public java.lang.String smiles()
      ```

      Returns:
      :   the smiles


    - #### nonisomericSmiles

      ```
      public java.lang.String nonisomericSmiles()
      ```

      Returns:
      :   non isomeric smiles


    - #### jmeFile

      ```
      public java.lang.String jmeFile()
      ```


    - #### reset

      ```
      public void reset()
      ```


    - #### clear

      ```
      public void clear()
      ```

      Same effect as clicking on the clear icon: clear the molecular drawing area


    - #### setTemplate

      ```
      public void setTemplate(java.lang.String templateAsJmeMol,
                              java.lang.String templateName)
      ```

      Provide a template, a molecular fragment with one marked atom, in JME format.

      Parameters:
      :   `templateAsJmeMol` -
      :   `templateName` -


    - #### setSubstituent

      ```
      public void setSubstituent(java.lang.String s)
      ```

      Select the next FG to be used. Deprecated because of the FG popup menu.

      Parameters:
      :   `s` -


    - #### repaint

      ```
      public void repaint()
      ```

      Redraw the whole applet if it is visible


    - #### deferredRepaint

      ```
      public void deferredRepaint()
      ```

      Repaint the applet after the browser event loop returns.
      TODO: this is not an applet.repaint() but a rerendering of all graphics objects
      See also `export.client.Utils.runAfterBrowserEventLoopReturns`


    - #### getHelpURL

      ```
      public java.lang.String getHelpURL()
      ```

      Returns:
      :   the url used to display JSME help page


    - #### setHelpURL

      ```
      public void setHelpURL(java.lang.String url)
      ```

      set the url to display the JSME help page. This method can be used to redefine the standard help page.

      Parameters:
      :   `url` -


    - #### alert

      ```
      public void alert(java.lang.String message)
      ```

      Display an alert box with the look and feel of JSME

      Parameters:
      :   `message` -


    - #### showInfo

      ```
      public void showInfo(java.lang.String message)
      ```

      Display a message in the bottom bar. Can be used to display e.g.the name or the MW of the compound in the editor.

      Parameters:
      :   `message` -


    - #### getNotifyStructuralChangeJSfunction

      ```
      @Deprecated
      public java.lang.String getNotifyStructuralChangeJSfunction()
      ```

      Deprecated.

      Returns:
      :   the notifyStructuralChangeJSfunction


    - #### setNotifyStructuralChangeJSfunction

      ```
      @Deprecated
      public void setNotifyStructuralChangeJSfunction(java.lang.String notifyStructuralChangeJSfunction)
      ```

      Deprecated.

      Specify a JS function that will be called each time the molecular structure in the editor has been changed.
      This method is deprecated. Use the `setAfterStructureModifiedCallback(JavaScriptObject)` method instead.

      Parameters:
      :   `notifyStructuralChangeJSfunction` - the name of the JavaScript function
          This method is deprecated. Use `setCallBack(String,JavaScriptObject)` instead.


    - #### setAfterStructureModifiedCallback

      ```
      @Deprecated
      public void setAfterStructureModifiedCallback(JavaScriptObject callbackFunction)
      ```

      Deprecated.

      Specify a JS function that will be called each time the molecular structure in the editor has been changed.
      The argument of the function will be a JSMEevent object that contains the fields: src (the jsme instance), the action, the atom index, the bond index and molecule index.
      Not all events have been mapped to a JSMEevent yet - action is then null.

      Parameters:
      :   `callbackFunction,` - a Javascript function - not the name of the function
          This method is deprecated. Use `setCallBack(String,JavaScriptObject)` instead.


    - #### setCallBack

      ```
      public void setCallBack(java.lang.String callbackName,
                              JavaScriptObject callbackFunction)
      ```

      Generic call back. The callback function is a JavaScript function that receives an instance of JSMEevent.
      See `getAlldefinedCallBackNames()` for all predefined callback names.

      Parameters:
      :   `callbackName` -
      :   `callbackFunction` -


    - #### getCallBack

      ```
      public JavaScriptObject getCallBack(java.lang.String callbackName)
      ```

      Generic callback

      Parameters:
      :   `callbackName` -

      Returns:
      :   the JavaScript function (can be null)


    - #### getAlldefinedCallBackNames

      ```
      public java.lang.String[] getAlldefinedCallBackNames()
      ```

      Return an array containing all possible callback names.


    - #### setAtomToHighLight

      ```
      public void setAtomToHighLight(int molIndex,
                                     int atomIndex)
      ```


    - #### setNotifyAtomHighLightChangeJSfunction

      ```
      @Deprecated
      public void setNotifyAtomHighLightChangeJSfunction(java.lang.String notifyAtomHighLightJSfunction)
      ```

      Deprecated.

      Specify a JS function that will be called each time the mouse pointer touches an atom.
      When the cursor leaves the atom, the atom index sent is 0.
      The argument is the name of a JavaScript function that receives two arguments: the molecule index and the atom index.
      To cancel, set the argument to null.
      Note valid atom indices are > 0.
      This method is deprecated. Use `setCallBack(String,JavaScriptObject)` instead.

      Parameters:
      :   `notifyAtomHighLightJSfunction` - must be a string


    - #### setAfterPasteCallback

      ```
      @Deprecated
      public void setAfterPasteCallback(JavaScriptObject callbackFunction)
      ```

      Deprecated.

      Specify a JavaScript function that will be called after a successful paste operation.
      Unlike the setNotify\* methods that have a string as an argument, this method requires a function.
      Example:
      jsmeApplet.setAfterPasteCallBack(function(jsmeEvent, pasteContent) {
      console.log(jsmeEvent.src..smiles());
      console.log(pasteContent);
      });
      This method might be removed in the future . Not supported by the pure Java applet JME.

      Parameters:
      :   `callbackFunction` - with two arguments, the jsme instance and the content that was pasted.
          This method is deprecated. Use `setCallBack(String,JavaScriptObject)` instead.


    - #### setBeforePasteCallback

      ```
      @Deprecated
      public void setBeforePasteCallback(JavaScriptObject callbackFunction)
      ```

      Deprecated.

      Specify a JavaScript function that will be called before a paste operation.
      Unlike the setNotify\* methods that have a string as an argument, this method requires a function.
      Example:
      jsmeApplet.setBeforePasteCallBack(function(jsmeEvent, pasteContent) {
      console.log(jsmeEvent.src.smiles());
      console.log(pasteContent);
      });
      Note that the paste operation into JSME is not performed.
      See the example shown in the file JSME\_chemical\_resolver\_demo.html
      It is not supported by the pure Java applet JME.

      Parameters:
      :   `callbackFunction` - with two arguments, the jsme instance and the content that was pasted.
          This method is deprecated. Use `setCallBack(String,JavaScriptObject)` instead.


    - #### setAtomBackgroundColors

      ```
      public void setAtomBackgroundColors(int molIndex,
                                          java.lang.String atomAndColorCSV)
      ```

      Parameters:
      :   `molIndex` -
      :   `atomAndColorCSV` - : at,c,at,c,at,c, ... at: atom index; c: color index


    - #### resetAtomColors

      ```
      public void resetAtomColors(int molIndex)
      ```

      Reset background colors

      Parameters:
      :   `molIndex` -


    - #### getPrePasteJSfunction

      ```
      public java.lang.String getPrePasteJSfunction()
      ```

      Returns:

      See Also:
      :   `setPrePasteJSfunction`


    - #### setPrePasteJSfunction

      ```
      @Deprecated
      public void setPrePasteJSfunction(java.lang.String prePasteJSfunction)
      ```

      Deprecated.

      Set a JS function that will be called before the paste is performed. This function will receive one argument: a string that a represent a molecular structure.
      The return value of the function is a string.
      Purpose: convert a name or a SMILES or an InchI into a MOL.

      Parameters:
      :   `prePasteJSfunction` - This method is deprecated. Use `setCallBack(String,JavaScriptObject)` instead.


    - #### getPasteLabel

      ```
      public java.lang.String getPasteLabel()
      ```

      See `setPasteLabel(String)`

      Returns:
      :   the currently used label.


    - #### setPasteLabel

      ```
      public void setPasteLabel(java.lang.String pasteLabel)
      ```

      Set the label of the context popup menu for pasting string, instead of using the default label.
      Purpose: This function can be used in case the paste action is customized with a callback function.
      See `setPrePasteJSfunction(String)`

      Parameters:
      :   `pasteLabel` -


    - #### getMolecularAreaLineWidth

      ```
      public float getMolecularAreaLineWidth()
      ```

      Returns:
      :   line width in pixel


    - #### setMolecularAreaLineWidth

      ```
      public void setMolecularAreaLineWidth(float molecularAreaLineWidth)
      ```

      Line width in pixel

      Parameters:
      :   `molecularAreaLineWidth` -


    - #### setAtomMolecularAreaFontSize

      ```
      public void setAtomMolecularAreaFontSize(float fs)
      ```


    - #### getMolecularAreaAntiAlias

      ```
      public boolean getMolecularAreaAntiAlias()
      ```

      Return the antialias setting for the molecular drawing area


    - #### setMolecularAreaAntiAlias

      ```
      public void setMolecularAreaAntiAlias(boolean molecularAreaAntiAlias)
      ```

      Set antialias for the molecular drawing area

      Parameters:
      :   `molecularAreaAntiAlias` -


    - #### setSize

      ```
      public void setSize(java.lang.String width,
                          java.lang.String height)
      ```

      Sets the object's size. This size does not include decorations such as
      border, margin, and padding.
      Setting the height to "100%" does not work.

      Parameters:
      :   `width` - the applet's new width, in CSS units (e.g. "10px", "1em", "100%")
      :   `height` - the applet's new height, in CSS units (e.g. "10px", "1em")


    - #### setSize

      ```
      public void setSize(int width,
                          int height)
      ```

      Sets the object's size. This size does not include decorations such as
      border, margin, and padding.
      Setting the height to "100%" does not work.

      Parameters:
      :   `width` - the applet's new width, in pixels
      :   `height` - the applet's new height, in pixels


    - #### setSize

      ```
      public void setSize(java.lang.String width,
                          java.lang.String height,
                          boolean resizeParent)
      ```

      Sets the object's size. This size does not include decorations such as
      border, margin, and padding.
      Setting the height to "100%" does not work.
      if resizeParent is true, then the parent HTML container (e.g. a DIV) in which the applet resides will
      be resized as well

      Parameters:
      :   `width` - the applet's new width, in CSS units (e.g. "10px", "1em", "100%")
      :   `height` - the applet's new height, in CSS units (e.g. "10px", "1em")


    - #### setSize

      ```
      public void setSize(int width,
                          int height,
                          boolean resizeParent)
      ```

      Sets the object's size. This size does not include decorations such as
      border, margin, and padding.
      Setting the height to "100%" does not work.
      if resizeParent is true, then the parent HTML container (e.g. a DIV) in which the applet resides will
      be resized as well

      Parameters:
      :   `width` - the object's new width, in pixels
      :   `height` - the object's new height, in pixels


    - #### setWidth

      ```
      public void setWidth(java.lang.String width)
      ```

      Sets the object's width. This width does not include decorations such as
      border, margin, and padding.

      Parameters:
      :   `width` - the object's new width, in CSS units (e.g. "10px", "1em")


    - #### setHeight

      ```
      public void setHeight(java.lang.String width)
      ```

      Sets the object's height. This height does not include decorations such as
      border, margin, and padding.

      Parameters:
      :   `height` - the object's new height, in CSS units (e.g. "10px", "1em")


    - #### getOffsetSize

      ```
      public JsArrayInteger getOffsetSize()
      ```

      Gets the object's offset width and height in pixels. This is the total width (height) of the object, including decorations such
      as border and padding, but not margin.

      Returns:
      :   an array of size 2 containing the width and height


    - #### getOffsetWidth

      ```
      public int getOffsetWidth()
      ```

      Gets the object's offset width in pixels. This is the total width of the object, including decorations such
      as border and padding, but not margin.


    - #### getOffsetHeight

      ```
      public int getOffsetHeight()
      ```

      Gets the object's offset height in pixels. This is the total height of the object, including decorations such
      as border and padding, but not margin.


    - #### setVisible

      ```
      public void setVisible(boolean visible)
      ```

      Shows or hides the given element. Also updates the "aria-hidden" attribute.

      Warning: implemented with a heuristic based on the "display" style:
      clears the "display" style to its default value if `visible` is true,
      else forces the style to "none". If the "display" style is set to "none"
      via CSS style sheets, the element remains invisible after a call to
      `setVisible(true)`.


    - #### isVisible

      ```
      public boolean isVisible()
      ```

      Returns whether the given element is visible in a way consistent with
      `setVisible(boolean)`.

      Warning: implemented with a heuristic. The value returned takes into
      account only the "display" style, ignoring CSS and Aria roles, thus may not
      accurately reflect whether the element is actually visible in the browser.


    - #### replaceAllAppletsByJSME

      ```
      public static int replaceAllAppletsByJSME()
      ```

      Scan the DOM for DIV's containing an attribute code="JME.class" and install a running JSME.

      Returns:
      :   the number of JSME installed.


    - #### replaceAllAppletsByJSME

      ```
      public static int replaceAllAppletsByJSME(JsClosure jsFunctionWithJsmeInstanceArg)
      ```

      Scan the DOM for DIV's containing an attribute code="JME.class" and install a running JSME. Call the argument when done.
      See also `getCreationIndex`.

      Parameters:
      :   `jsFunction:` - a function defined in JavaScript such as : function(eachJsmeInstance){alert("JSME created. Creation index: "+ eachJsmeInstance.getCreationIndex() )};

      Returns:
      :   the number of new JSME installed.


    - #### addClickHandler

      ```
      public void addClickHandler(JsClosureJsme jsEventHandler)
      ```

      Add a click handler to a JSME instance. The argument is a JavaScript function with two arguments:
      - the JSME instance that received the click event.
      - the mouse event
      Example:
      document.JSME.addClickHandler(function(jsme, evt) {
      alert("My smiles is: " + jsme.smiles());
      });
      Purpose: intercept a click event on JSME in depict mode.

      Parameters:
      :   `jsEventHandler:` - a JavaScript function with two arguments.


    - #### addMouseDownHandler

      ```
      public void addMouseDownHandler(JsClosureJsme jsEventHandler)
      ```

      Add a mouse down handler to a JSME instance. The argument is a JavaScript function with two arguments:
      - the JSME instance that received the click event.
      - the mouse event
      Example:
      document.JSME.addMouseDownHandler(function(jsme, evt) {
      alert("My smiles is: " + jsme.smiles());
      });
      Purpose: intercept a click event on JSME in depict mode.

      Parameters:
      :   `jsEventHandler:` - a JavaScript function with two arguments.


    - #### addMouseOverHandler

      ```
      public void addMouseOverHandler(JsClosureJsme jsEventHandler)
      ```

      Add a mouse over handler to a JSME instance. The argument is a JavaScript function with two arguments:
      - the JSME instance that received the event.
      - the mouse event
      Example:
      document.JSME.addMouseOverHandler(function(jsme, evt) {
      console.log("My smiles is: " + jsme.smiles());
      });
      Purpose: intercept a mouse event on JSME in depict mode.

      Parameters:
      :   `jsEventHandler:` - a JavaScript function with two arguments.


    - #### addMouseOutHandler

      ```
      public void addMouseOutHandler(JsClosureJsme jsEventHandler)
      ```

      Add a mouse out handler to a JSME instance. The argument is a JavaScript function with two arguments:
      - the JSME instance that received the event.
      - the mouse event
      Example:
      document.JSME.addMouseOutHandler(function(jsme, evt) {
      console.log("My smiles is: " + jsme.smiles());
      });
      Purpose: intercept a mouse event on JSME in depict mode.

      Parameters:
      :   `jsEventHandler:` - a JavaScript function with two arguments.


    - #### setAfterDepictEditToggleEventHandler

      ```
      public void setAfterDepictEditToggleEventHandler(JsClosureJsme jsEventHandler)
      ```


    - #### setAction

      ```
      public void setAction(int action)
      ```

      select one of the menu action without having to click on the menu.
      Purpose: enable some action in depict mode, e.g. the atom selection with the star option.
      Example : 105 is the code for ACTION\_MARK.
      In theory, it is possible to program a new menu interface for JSME using all action codes.

      Parameters:
      :   `action` -


    - #### isDepictMode

      ```
      public boolean isDepictMode()
      ```

      Returns:
      :   true if the editor is in depict mode


Skip navigation links


- Package
- Class
- Tree
- Deprecated
- Index
- Help

- Prev Class
- Next Class

- Frames
- No Frames

- All Classes

- Summary:
- Nested |
- Field |
- Constr |
- Method

- Detail:
- Field |
- Constr |
- Method
